# Quality of end-of-life care in general practice – a pre–post comparison of a two-tiered intervention

**DOI:** 10.1186/s12875-022-01689-9

**Published:** 2022-04-20

**Authors:** Katharina van Baal, Birgitt Wiese, Gabriele Müller-Mundt, Stephanie Stiel, Nils Schneider, Kambiz Afshar

**Affiliations:** Institute for General Practice and Palliative Care, Hannover Medical School, Carl-Neuberg-Straße 1, 30625 Hannover, Germany

**Keywords:** General practice, Primary care, End-of-life care, Palliative care, Health services research, Quality of health care

## Abstract

**Background:**

General practitioners (GPs) play a crucial role in the provision of end-of-life care (EoLC). The present study aimed at comparing the quality of GPs’ EoLC before and after an intervention involving a clinical decision aid and a public campaign.

**Methods:**

The study was part of the larger interventional study ‘Optimal care at the end of life’ (OPAL) (Innovation Fund, Grant No. 01VSF17028). The intervention lasted 12 months and comprised two components: (1) implementation of the Supportive and Palliative Care Indicators Tool (SPICT-DE™) in general practice and (2) a public campaign in two German counties to inform and connect regional health care providers and stakeholders in EoLC. Participating GPs completed the General Practice End of Life Care Index (GP-EoLC-I) pre- (t0) and post- (t1) intervention. The GP-EoLC-I (25 items, score range: 14–40) is a self-assessment questionnaire that measures the quality of GPs’ EoLC. It includes two subscales: practice organisation and clinical practice. Data were analysed descriptively, and a paired t-test was applied for the pre–post comparison.

**Results:**

Forty-five GPs (female: 29%, median age: 57 years) from 33 general practices participated in the intervention and took part in the survey at both times of measurement (t0 and t1). The mean GP-EoLC-I score (t0 = 27.9; t1 = 29.8) increased significantly by 1.9 points between t0 and t1 (t(44) = − 3.0; *p* = 0.005). Scores on the practice organisation subscale (t0 = 6.9; t1 = 7.6) remained almost similar (t(44) = -2.0; *p* = 0.057), whereas those of the clinical practice subscale (t0 = 21.0; t1 = 22.2) changed significantly between t0 and t1 (t(44) = -2.6; *p* = 0.011). In particular, items regarding the record of care plans, patients’ preferred place of care at the end of life and patients’ preferred place of death, as well as the routine documentation of impending death, changed positively.

**Conclusions:**

GPs’ self-assessed quality of EoLC seemed to improve after a regional intervention that involved both the implementation of the SPICT-DE™ in daily practice and a public campaign. In particular, improvement related to the domains of care planning and documentation.

**Trial registration:**

The study was registered in the German Clinical Trials Register (DRKS00015108; 22/01/2019).

## Background

### Palliative care needs at the end of life

International data suggest that approximately 75% of all people at the end of life are in need of palliative care (PC) [1]. In 2019, approximately 940,000 people died in Germany [2]. In the German context, outpatient PC is commonly separated into generalist and specialist outpatient PC [3, 4]. Generalist outpatient PC for patients in the community is mostly provided for patients with a relatively low symptom intensity [3]. Specialist outpatient PC is usually provided by interdisciplinary teams for patients with higher symptom burden and complexity [4]. The vast majority of people at the end of life can be cared for within the framework of generalist outpatient PC, which is typically provided by general practitioners (GPs) [3]. Thus, GPs play a key role in outpatient PC and end-of-life care (EoLC) [5, 6].

### Quality of end-of-life care

Quality indicators are regularly applied to measure the quality of EoLC [7–10]. In Germany, the number of patients treated within specialist outpatient PC has increased in recent years, while the provision and remuneration of generalist outpatient PC is consistent or even declining [11, 12]. In addition, generalist outpatient PC tends to be provided relatively late in the care trajectory, mainly in the last weeks prior to death [11]. It is well known that PC should be considered early in the care trajectory [13]. An early initiation of PC may improve several patient-oriented outcomes at the end of life, such as symptom burden and quality of life [14, 15]. An early identification also seems to improve outcomes for carers, such as coordination and teamwork [16, 17]. In addition to the identification of PC needs, the early initiation of PC actions such as conversations about death and dying, preparation of advance directives or health care proxies for the purpose of advance care planning (ACP) is challenging [18, 19].

However, the early identification of patients with PC needs is particularly difficult for GPs in daily practice due to, among other reasons, prognostic uncertainty [20–22]. In this context, the Supportive and Palliative Care Indicators Tool (SPICT™) [23] can support the systematic identification of patients with PC needs. A version of this tool is available for the German-speaking area (SPICT-DE™) [24], and it was applied as part of the intervention in the present study. Additionally, the study included the General Practice End of Life Care Index (GP-EoLC-I), which is a specific measure of the quality of GPs’ EoLC [25, 26].

### Preliminary work

The ‘Optimal care at the end of life’ (OPAL) study is a Medical Research Council (MRC) phase-I study for complex interventions [27]. Employing a mixed-methods and pre–post design, the study aims at improving the provision of outpatient PC by GPs and health care providers for patients in their last phase of life [28]. Phase 1 of the OPAL study assessed standard PC provision in two counties in Lower Saxony (baseline, t0) [28]. A key component of this research was a standardised written survey of GPs to evaluate the quality of their EoLC from their own perspectives [29]. The t0 results indicated potential for improvement regarding the systematic identification of patients with potential PC needs, the realisation of multi-disciplinary case conferences to discuss patients with PC needs, the routine application of care protocols and symptom assessment tools, the documentation of patients’ wishes and beliefs around EoLC, and the inclusion of informal caregivers [29]. GPs highlighted coordination and cooperation between stakeholders in EoLC as the most relevant indicators of good PC [29]. Furthermore, they identified the use of standardised tools (e.g. those used to systematically identify patients with potential PC needs) as an important requirement for improving the quality of PC [29].

Phase 2 included a regional intervention, which implemented both the SPICT-DE™ [24, 30] in general practices and a public campaign to connect stakeholders and health care providers involved in EoLC [28].

### Research gap, objective and research questions

In phase 3, we evaluated whether the implementation of the SPICT-DE™ to support the identification of patients with potential PC needs in general practice and a public campaign to improve collaboration and cooperation between stakeholders had an impact on the quality of EoLC provided by GPs. Therefore, the standard provision of PC was re-assessed (follow-up, t1) to facilitate a comparison between t0 and t1 and to investigate the potential effects of the intervention in phase 3 of the OPAL study [28].

The main objective of the present study was to compare the quality of GPs’ EoLC before and after the intervention. Specifically, the following research questions were addressed:

- How did the two-tiered regional intervention influence the quality of GPs’ EoLC?
- Which items of the GP-EoLC-I showed significant differences in the pre–post comparison?

## Methods

### Study design

The present study was part of OPAL [28] – a prospective interventional mixed-methods study with a pre–post design. The study follows the MRC guidance for developing and evaluating complex interventions [31].

### Setting

Due to its regional approach, OPAL was conducted in two counties in Lower Saxony, Germany. Both counties are so-called ‘local health regions’ with a special interest in facilitating cooperation between health care providers [32].

### Study population and recruitment

The main study population consisted of practicing GPs in both counties in Lower Saxony.

In October 2018, all registered GPs in both counties (*n* = 190 GPs in *n* = 124 general practices), excluding those only treating privately insured patients, were invited to take part in the study. Requests were maintained via phone, letter and fax until a response was recorded by each general practice. During the recruitment, brief and clear study information was sent to the general practices – if necessary, repeatedly. The recruitment phase ended in April 2019 [29].

Additionally, clinical data regarding patients with chronic, progressive disease (aged ≥18 years and with statutory health insurance) who died between April and September 2018 (t0) or 2020 (t1) while receiving medical treatment by the participating GPs were gathered.

### Main outcome: GP-EoLC-I

A standardised written survey of the quality of GPs’ EoLC was administered at t0 (between October 2018 and May 2019) [29] and t1 (between October 2020 and March 2021), using the German version of the GP-EoLC-I (i.e. the main target variable in OPAL). The GP-EoLC-I was developed at the University of Sheffield [25] and systematically translated, tested and adjusted for the German context by the Institute for General Practice and Palliative Care at Hannover Medical School [26].

The GP-EoLC-I measures GPs’ perceptions of the quality of their PC and EoLC [25]. Scores for two subscales, practice organisation (12 items; score range: 1–14) and clinical care (13 items; score range: 13–26), are summarised to create an index score (25 items, score range 14–40), with higher scores indicating higher quality of EoLC. The original version of the GP-EoLC-I was found to demonstrate satisfactory internal consistency and reliability [25]. More details regarding the GP-EoLC-I and its German version can be found elsewhere [25, 26]. To collect additional data on the participating general practices (i.e. structural characteristics), a semi-structured questionnaire was also administered. For each general practice, the questionnaire was completed both before (t0) and after (t1) the intervention.

### Clinical data

Medical assistants or GPs collected clinical data pertaining to the deceased patients, using their electronic or analogue documentation systems. The provision of generalist and specialist outpatient PC is in Germany defined by the use of specific remuneration digits, which were the basis for this analysis. In addition to patients’ sociodemographic data (i.e. age, sex, date of death, diagnoses in the four quarters prior to death), the following data regarding the provision of generalist and specialist PC were assessed:

- number of patients receiving generalist outpatient PC in the last year of life;
- onset of generalist outpatient PC prior to death (in days);
- number of patients receiving specialist outpatient PC in the last year of life; and
- onset of specialist outpatient PC prior to death (in days).

The results regarding the clinical data assessed at t0 (with respect to patients who died between April and September 2018) have been published elsewhere [33]. In this article, the clinical data of patients who died between April and September 2020 (t1) are used to describe the patient population treated in the general practices, in comparison to t0 data.

### Intervention

The intervention lasted 12 months (from April/May 2019 to April/May 2020) and comprised two components: (1) the implementation of the SPICT-DE™ in general practice and (2) a public campaign to inform and connect EoLC health care providers and stakeholders in both counties.

1. Intervention in general practices (i.e. SPICT-DE™)

The SPICT™ is a clinical tool that was developed in 2010 by the Primary Palliative Care Research Group at the University of Edinburgh, as part of the Gold Standards Framework in the United Kingdom [25]. It aims at supporting the identification of patients with deteriorating health and potentially unmet PC needs [24, 34]. The German version of the SPICT™ was systematically translated and adjusted by the Institute for General Practice and Palliative Care at Hannover Medical School [24]. Furthermore, it was tested in two pilot studies and proved to be a feasible and practical tool supporting the systematic identification if patients with PC needs [34, 35].

Each participating GP received standardised training in using the SPICT-DE™, alongside an in-depth definition of the term ‘palliative care’, in an appointment lasting approximately 30 min. In more detail, the user training conveyed information on the aim and background of using the SPICT-DE™ (according to the published guide) [36]; the intervention process, itself; when and how to use the SPICT-DE™; and the documentation and inclusion and exclusion criteria for patients to be assessed with the SPICT-DE™. The user training was carried out by two scientific research assistants under a physician’s supervision.

After completing the user training, GPs received a manual containing the most important information. They were asked to apply the SPICT-DE™ in their daily practice for a duration of 12 months, with every patient aged ≥18 years with chronic, progressive disease, seen in the practice or in the patient’s home. Patients who were already receiving specialist outpatient PC and hospice residents were excluded. During the intervention phase, the study team scheduled three monitoring visits with reflection talks in each general practice every 4 months, to observe the implementation of the SPICT-DE™ and to discuss emerging questions. The third monitoring visit was simultaneous with the end of the intervention phase.

(2) Health region intervention (i.e. public campaign)

To inform EoLC stakeholders and health care providers in both counties, the study intervention implemented a public campaign. Through flyers, newsletters and articles published in the local press, the study team informed stakeholders about the study aims and the implementation of the SPICT-DE™ in general practices. Furthermore, a multidisciplinary discussion panel (“health dialogue”) was held in February 2020, aimed at the development of strategies to improve EoLC and better connect scientific work and practice in both regions. The study team documented all forms of contact being made during the public campaign and recorded results and insights of the multidisciplinary discussion panel.

### Data analysis

Based on the recruitment rates of previous studies, a sample size was calculated in advance of recruitment [28]. A power analysis detected a medium effect of the intervention for a sample of 50 GPs. Furthermore, a sample size of 50 GPs was determined to have approximately 80% power to indicate an average difference of four points in the comparison between t0 and t1 (10 points standard deviation (SD) assumed; paired t-test, two-sided significance level of 5%) [25, 28]. Data were analysed using version 26 of the Statistical Package for Social Sciences (SPSS Inc., Chicago, IL/USA). Descriptive analyses included the calculation of means, SDs, medians and interquartile ranges (IQR). Clinical data of the deceased patients were analysed descriptively. Missing values were not replaced. Smaller sample sizes were stated for all items, indicating missing values. The 25 items comprising the GP-EoLC-I score contained no missing data. A paired t-test was used for the pre–post comparison of the GP-EoLC-I score, subscale scores and single item scores. Data regarding GPs who dropped out between timepoints were excluded from the analysis.

### Ethics and data protection

The study was approved by the Ethics Committee of the Hannover Medical School in August 2018 (No.: 8038_BO_K_2018) and all methods were performed in accordance with the Declaration of Helsinki. Only pseudonymised data were analysed. The present study followed the data security procedure described in the study protocol of the main study OPAL [28].

## Results

**Fig. 1:**
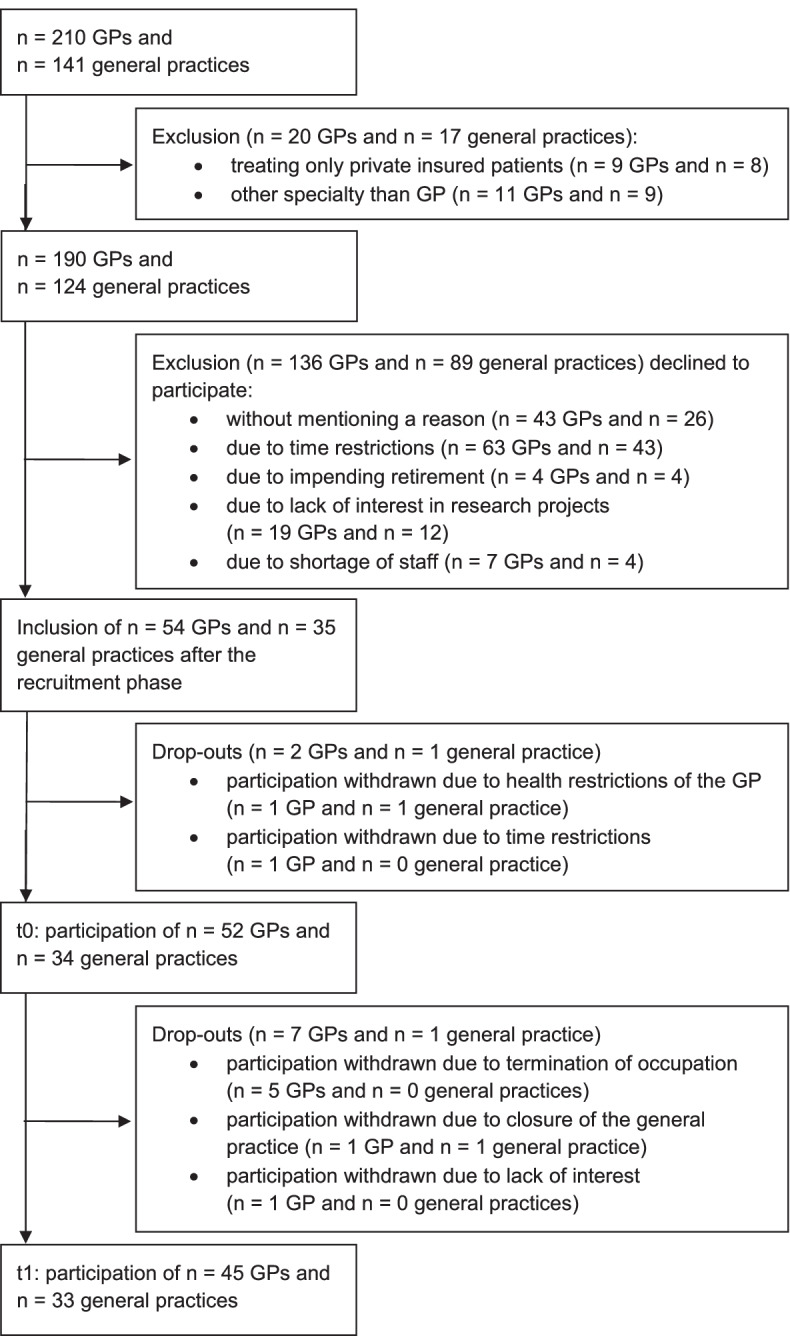
Flow chart describing the inclusion/exclusion of general practices and general practitioners (supplementing Fig. 1 [29])

At t0, 52 GPs from 34 general practices participated in the study (recruitment rate of 27.4% of all eligible general practices). At t1, 45 GPs from 33 general practices took part in the post-intervention survey (Fig. 1).

### Description of the study sample

A total of 45 GPs from 33 general practices participated in the survey at both t0 and t1. The following description of the study sample refers to data assessed at t1. Thirty-two male (71.1%) and 13 female (28.9%) GPs were included in the study sample. Participating GPs were aged 31–79 years with a mean age of 55 years (*n* = 44; SD = 10.4). More than half of the GPs worked in single practices (51.1%). The median duration of their clinical practice was 28 years (*n* = 41; IQR = 20.0–32.0) as a physician and 20 years (IQR = 11.0–25.5) as a GP. Table 1 presents further information on the study sample.

**Table 1:** Description of the study sample (*N* = 45 GPs)

Variable		n	%
Practice form	Single practice	23	51.1
	Group practice	18	40.0
	Joint practice	3	6.7
	Medical care centre	1	2.2
Care region	Medium-sized city	18	40.0
	Small town	13	28.9
	Rural community	14	31.1
Part of a teaching practice	Yes	15	33.3
	No	30	66.7
Palliative care qualification^*^ (multiple responses possible)	Basic course	15	33.3
	Additional qualification (incl. basic course)	9	20.0
	Other qualification (e.g. experience on a PC ward)	7	15.6
	None	22	48.9
Activity in a palliative care initiative^*^ (multiple responses possible)	Hospice association	5	11.1
	Quality circle	8	17.8
	Palliative network	4	8.9
	Specialist outpatient palliative care team	11	24.4
	Other initiative	2	4.4
	None	26	57.8

*PC* palliative care, ^*^number of participants confirming this detail

### Clinical data of the deceased patients

At t0, data pertaining to 302 deceased patients (48% female) from 32 general practices were included in the analyses. Patients’ median age at death was 82 years (IQR = 73–87; *n* = 300). The t1 analyses included data pertaining to 154 deceased patients (54.5% female; median age at death: 84 years; IQR = 77–89) from 23 general practices. Table 2 displays the EoLC indicators based on the clinical data of the deceased patients, describing the patient population treated in the participating general practices.

**Table 2:** Provision and onset of generalist and specialist palliative care for patients in the participating general practices who died in 2018 and 2020

Indicator		t0		t1	
		n	%	n	%
Number of patients receiving generalist outpatient PC (t0: *N* = 302; t1: *N* = 154)	yes	85	28.1	33	21.4
	no	215	71.2	117	76.0
	missing value	2	0.7	4	2.6
Onset of generalist outpatient PC prior to death (in days) (t0: *N* = 85; t1: *N* = 33)	0–3	10	11.8	3	9.1
	4–10	13	15.3	3	9.1
	11–20	11	12.9	3	9.1
	21–30	7	8.2	0	0.0
	31–60	9	10.6	3	9.1
	61–120	7	8.2	3	9.1
	121–240	5	5.9	5	15.1
	≥241	10	11.8	4	12.1
	missing value	13	15.3	9	27.3
Number of patients receiving specialist outpatient PC (t0: *N* = 302; t1: *N* = 154)	yes	56	18.5	35	22.7
	no	241	79.8	115	74.7
	missing value	5	1.7	4	2.6
Onset of specialist outpatient PC prior to death (in days)* (t0: *N* = 56; t1: *N* = 35)	0–3	4	7.1	3	8.6
	4–10	12	21.4	7	20.0
	11–20	4	7.1	2	5.7
	21–30	9	16.1	2	5.7
	31–60	8	14.3	3	8.6
	61–120	10	17.9	5	14.3
	121–240	3	5.4	0	0.0
	≥241	3	5.4	5	14.3
	missing value	3	5.4	8	22.9

*PC* palliative care; *t0* pre-intervention; *t1* post-intervention; *differences due to rounding

### GP-EoLC-I: descriptive analyses

The mean GP-EoLC-I score at t0 was 27.9 (SD = 4.2) and, at t1, 29.8 (SD = 4.2), considering the 45 GPs who participated at both t0 and t1. The practice organisation subscale scores (Table 3) had a mean value of 6.9 at t0 (SD = 2.1) and 7.6 at t1 (SD = 2.3). The clinical care subscale scores (Table 4) had a mean value of 21.0 at t0 (SD = 3.1) and 22.2 at t1 (SD = 2.5). Tables 3 and 4 display the results for each item of the two subscales, comparing data between t0 and t1.

**Table 3:** GP-EoLC-I practice organisation subscale items [25, 26] at t0 and t1 (*N* = 45 GPs)

Item		t0		t1	
		n	%	n	%
Systematic identification in the case file	Never	21	46.7	13	28.9
	Sometimes	11	24.4	11	24.4
	Mostly	8	17.8	16	35.6
	Always	5	11.1	5	11.1
		**Yes [n (%)]**	**No [n (%)]**	**Yes [n (%)]**	**No [n (%)]**
Inclusion criteria for PC register	Cancer diagnosis	37 (82.2)	8 (17.8)	40 (88.9)	5 (11.1)
	Life-limiting non-malignant disease	35 (77.8)	10 (22.2)	44 (97.8)	1 (2.2)
	Terminal disease	43 (95.6)	2 (4.4)	45 (100.0)	0 (0.0)
	Increasing need for nursing and help in everyday life	11 (24.4)	34 (75.6)	14 (31.1)	31 (68.9)
	None of these	1 (2.2)	44 (97.8)	0 (0.0)	45 (100.0)
Multi-disciplinary forum for discussing PC patients	Formal regular meeting	3 (6.7)	42 (93.3)	7 (15.6)	38 (84.4)
	Formal occasional meeting	3 (6.7)	42 (93.3)	9 (20.0)	36 (80.0)
	Informal regular discussions	6 (13.3)	39 (86.7)	10 (22.2)	35 (77.8)
	Ad hoc liaison	28 (62.2)	17 (37.8)	25 (55.6)	20 (44.4)
	None of these	11 (24.4)	34 (75.6)	7 (15.6)	38 (84.4)
System for coordinating PC		15 (33.3)	30 (66.7)	17 (37.8)	28 (62.2)
Named coordinator for PC		6 (13.3)	39 (86.7)	7 (15.6)	38 (84.4)
Unified regional record of PC patients		13 (28.9)	32 (71.1)	10 (22.2)	35 (77.8)
System to ensure 24 h availability of anticipatory med.		35 (77.8)	10 (22.2)	38 (84.4)	7 (15.6)
Use of a protocol for the care of dying cancer patients		14 (31.1)	31 (68.9)	13 (28.9)	32 (71.1)
Use of a symptom assessment tool for PC patients		5 (11.1)	40 (88.9)	6 (13.3)	39 (86.7)

*med* medication, *PC* palliative care, *t0* pre-intervention, *t1* post-intervention

**Table 4:** GP-EoLC-I clinical care subscale items [25, 26] at t0 and t1 (*N* = 45 GPs)

	t0 n (%)				t1 n (%)			
Item	Always	Mostly	Sometimes	Rarely/never	Always	Mostly	Sometimes	Rarely/never
Record care plans for PPC*	15 (33.3)	15 (33.3)	6 (13.3)	9 (20.0)	23 (51.1)	17 (37.8)	2 (4.4)	3 (6.7)
Encourage PPC in preparing for death in an active manner*	12 (26.7)	21 (46.7)	8 (17.8)	4 (8.9)	14 (31.1)	21 (46.7)	10 (22.2)	0 (0.0)
Assist PPC by addressing unfinished business	7 (15.6)	29 (64.4)	5 (11.1)	4 (8.9)	12 (26.7)	20 (44.4)	13 (28.9)	0 (0.0)
Assist PPC by preparing advance directives*	16 (35.6)	19 (42.2)	8 (17.8)	2 (4.4)	10 (22.2)	29 (64.4)	6 (13.3)	0 (0.0)
Record PPC wishes or spiritual beliefs	8 (17.8)	13 (28.9)	10 (22.2)	14 (31.1)	12 (26.7)	13 (28.9)	11 (24.4)	9 (20.0)
Record preferred place of care at the end of life / death*	9 (20.0)	11 (24.4)	9 (20.0)	16 (35.6)	15 (33.3)	14 (31.1)	10 (22.2)	6 (13.3)
Routinely assess and discontinue inappropriate interventions (incl. med.)	26 (57.8)	16 (35.6)	1 (2.2)	2 (4.4)	27 (60.0)	18 (40.0)	0 (0.0)	0 (0.0)
Record of a named family carer for discussion and coordination of care*	23 (51.1)	15 (33.3)	3 (6.7)	4 (8.9)	23 (51.1)	20 (44.4)	2 (4.4)	0 (0.0)
Disseminate appropriate written information to family and carers	4 (8.9)	5 (11.1)	11 (24.4)	25 (55.6)	4 (8.9)	12 (26.7)	18 (40.0)	11 (24.4)
Document the family’s or carers’ insights into the patient’s condition	4 (8.9)	18 (40.0)	13 (28.9)	10 (22.2)	8 (17.8)	17 (37.8)	11 (24.4)	9 (20.0)
Dispatch a handover form for out-of-hours care for PPC*	11 (24.4)	15 (33.3)	13 (28.9)	6 (13.3)	11 (24.4)	17 (37.8)	11 (24.4)	6 (13.3)
Out-of-hours availability to PPC in terminal stages of illness	17 (37.8)	12 (26.7)	11 (24.4)	5 (11.1)	18 (40.0)	12 (26.7)	9 (20.0)	6 (13.3)
Routine documentation of impending death*	8 (17.8)	11 (24.4)	11 (24.4)	15 (33.3)	13 (28.9)	14 (31.1)	6 (13.3)	12 (26.7)

*med* medication, *PPC* patients with palliative care, *t0* pre-intervention, *t1* post-intervention; *differences due to rounding

### GP-EoLC-I: comparison between t0 and t1

A statistical comparison between t0 and t1 using the paired t-test showed a significant increase between timepoints for the GP-EoLC-I score, with a mean difference of 1.9 points (t(44) = -3.0, *p* = 0.005). Additionally, the clinical care subscale score differed significantly between t0 and t1, with a mean alteration of 1.2 points (t(44) = -2.6, *p* = 0.011); and the practice organisation subscale score varied insignificantly between timepoints, with a mean difference of 0.7 points (t(44) = -2.0, *p* = 0.057). In particular, items regarding the record of care plans, patients’ preferred place of care at the end of life and patients’ preferred place of death, as well as the routine documentation of impending death, showed positive changes (Table 4).

### Public campaign and ‘health dialogue’

During the regional intervention, three newsletters were sent to participating GPs, stakeholders and health care providers at the interfaces of general practice. These newsletters included information on the progression of the study OPAL as well as ongoing and upcoming phases and tasks for the participants. In autumn 2019, the project, its aims and the intervention in general practices were presented at public health events in both counties. The multidisciplinary discussion panel ‘health dialogue’ took place in February 2020, with 36 participants. Several GPs and their practice teams, regional PC stakeholders (e.g. staff of hospices, specialised PC services, inpatient PC units, nursing services) and political representatives of both health regions took part in the event. The 4-h panel comprised two parts: (1) presentation of the OPAL phase 1 study results (i.e. evaluation phase t0) and (2) workshops with participants to discuss the results, develop strategies to improve EoLC, better link scientific work and practice and facilitate collaboration and cooperation of health care providers and stakeholders in both regions. Four workshops were held in groups with 6 to 10 participants and moderated by one member of the study team. Each participant attended two workshops. The content of the workshops related to key subjects of the results in phase 1: integration of relatives (1), identification of PC needs (2), cooperation and responsibilities (3) and change in awareness in the society (4). Participants received an information folder including a laminated version of SPICT-DE™.

Additionally, a flyer was sent to all participants in OPAL with information on the results of study phases 1 and 2 including the findings of the ‘health dialogue’. The public campaign aimed at optimising collaboration between health care providers and PC professionals, and the positive feedback and responses of stakeholders suggest that the campaign was successful.

## Discussion

The present study aimed at comparing the quality of GPs’ EoLC before and after an intervention in two counties in Lower Saxony, Germany, that included: (1) the implementation of the SPICT-DE™ in general practice as a clinical decision aid in identifying potential PC patients and (2) a public campaign to inform and connect EoLC stakeholders. To the best of our knowledge, OPAL is the first study to have assessed a regionwide implementation of the SPICT-DE™ in general practices in Germany. According to the GP-EoLC-I, GPs’ self-assessed quality of EoLC significantly improved after the intervention. In particular, items associated with the clinical care subscale regarding the record of care plans, patients’ preferred place of care at the end of life and patients’ preferred place of death, as well as the routine documentation of impending death, changed positively.

Compared to data from the United Kingdom in 2010 (mean 31.0) [25], GPs in our sample showed a lower GP-EoLC-I at t0 (with a difference of 3.1 points). After the intervention (t1), German GP scores approached those of the UK sample.

GPs often face difficulty identifying the adequate timepoint in a patient’s disease trajectory to initiate PC. This difficulty is especially pronounced in the case of patients with chronic, non-malignant disease [20, 23]. Although it is widely accepted that the timely identification of PC needs benefits patients [23, 37], the systematic identification of patients with potential PC needs in Germany remains inconsistent [7, 29].

Our results confirm earlier findings from different settings and populations, which identify the SPICT™ as a practical and helpful tool to support the identification of patients who might benefit from PC [24, 34, 38, 39]. The SPICT-DE™ might also improve GPs’ EoLC competencies and increase their awareness of the PC needs in general and particularly of patients with chronic non-malignant disease [24]. Significant differences between t0 and t1 were especially seen for items regarding the documentation of care plans. The SPICT-DE™ facilitates the administration and documentation of care plans, as well as the documentation of patients’ preferred place of care at the end of life and preferred place of death. These results emphasise the importance of patient-centred care and ACP.

It is crucial for health care providers to be confident in their decision making around ACP, in order to ensure a high quality of care [40, 41]. Advance care plans aim at bringing patients’ preferences in line with EoLC [42]. Accordingly, they may contribute to optimising the quality of EoLC [41]. However, ACP documents are not regularly available when needed, and several barriers to ACP have been acknowledged [18, 42, 43]. In particular, patients might avoid talking about EoLC for various reasons (e.g. a lack of knowledge, misleading interpretation of the relevance of ACP) [43]. GPs often have very personal and long-standing relationships with patients, and are therefore highly eligible to address EoLC topics [43].

Previous studies have revealed major challenges in GPs’ provision of EoLC. These include a need for more collaborative care and a lack of significant communication and cooperation between caregivers, stakeholders and patients [6, 29, 44, 45]. The present study addressed collaboration and cooperation by conducting a public campaign and connecting health care providers and stakeholders. The results might emphasise the importance of applying a two-tiered intervention, involving both GPs and regional stakeholders – underlining that the isolated clinical implementation of the SPICT-DE™ may not be sufficient to produce significant change. Thoonsen et al. [46] found no difference between the intervention and control group after a training in identifying patients in need of PC and anticipatory care planning. These results might underline the necessity for a multi-layered intervention. However, the present study identified room for further improvement after the intervention, particularly with respect to communication and co-ordination, which represent two of the so-called ‘seven Cs’ in the Gold Standards Framework for primary care [25, 47]. Thus, further steps to enhance GPs’ EoLC may include the early integration of health care professionals to coordinate EoLC and the determination of an employee responsible for such coordination, as described in the GP-EoLC-I practice organisation subscale. Also, close cooperation between GPs, specialised outpatient PC teams and other services might improve GPs’ EoLC [48, 49] and positively influence patient outcomes [50].

The inclusion of family caregivers in the provision of care is essential, as informal caregivers often provide the majority of EoLC [51, 52]. The GP-EoLC-I underlines the importance of informal caregivers, as three items on the clinical care subscale address their inclusion in generalist PC: recording a named family carer to discuss and coordinate care, disseminating appropriate written information to family members and carers, and documenting family members’ (and/or carers’) insights into the patient’s condition. Unfortunately, these items demonstrated potential for improvement at t0 and no significant improvement at t1. GPs support for family caregivers therefore represents a highly important field of action to improve GPs’ EoLC, and this should be addressed in future research.

To implement the stated fields of action in generalist PC (i.e. the identification of patients with potential PC needs, communication, cooperation, the inclusion of family members and carers), GPs require appropriate working conditions. The high effort associated with GPs’ daily practice of generalist outpatient PC is not sufficiently acknowledged in their remuneration. This represents a major barrier for the provision of generalist outpatient PC and the identification of PC needs [49]. Furthermore, time constraints and staff shortages represent additional obstacles to the provision of PC by GPs [53, 54]. These circumstances may contribute to the fact, that after the intervention in this study, an even smaller group of patients received generalist PC and the number of patients receiving either form of outpatient PC remained low. This results aligns with earlier findings from our research group [33, 55] and others [12]. Reasons for the overall low number of patients receiving generalist PC (and forms of outpatient PC in general) are heterogeneous. The already mentioned aspects regarding working conditions, staff shortage and remuneration might be contributing factors. The authors suggest, that the lack of concrete criteria for conducting generalist palliative care and inverted incentives on level of renumeration also play a role [56, 57]. Geriatric remuneration models or rates for patients with chronic diseases might simply be financially more attractive. Future research should address these factors and develop further strategies to improve the structural, legal and financial conditions for generalist PC in Germany [49]. A first step towards improvement might be the remuneration for applying the SPICT-DE™ in Germany.

The regional implementation strategy and the intervention applied in OPAL might be transferred to other regions, to Lower Saxony at large and to other federal states. In order to facilitate this, the study results should be included in medical education as well as residency and specialised PC training for physicians and other health care professionals [28].

## Strengths and limitations

The present study represented the first attempt to use the GP-EoLC-I to compare the quality of GPs’ EoLC before and after an intervention in Germany. Future studies might apply it to evaluate the quality of PC over time.

The results of the study relate to a selected region in Lower Saxony and cannot, therefore, be generalised unreservedly. The lack of a control group is a second important limitation, as it prevents us from concluding without reservation that the differences in the quality of GPs’ EoLC were (only) caused by the intervention. Furthermore, a selection bias might have been present, to the extent that participating GPs may have had greater interest in PC, and their insights may have therefore differed from those of the collective group of GPs in Germany. Furthermore, EoLC quality was self-assessed only by the participating GPs, and no insights from patients or relatives were included.

Although the pre–post comparison of the GP-EoLC-I showed statistically significant differences, the clinical significance remains questionable. Further analyses within the OPAL study will focus on the perspectives of PC experts and the relatives of deceased patients in general practice [28].

## Conclusions

The quality of GPs’ EoLC seemed to improve after a two-tiered regional intervention including: (1) the implementation of a clinical decision aid for the identification of patients with potential PC needs in general practice and (2) a public campaign to inform and connect EOLC providers. The GP-EoLC-I is effective at assessing and comparing the quality of GPs’ EoLC, as well as identifying potential areas for improvement.

## Data Availability

The datasets generated and/or analysed during the current study are not publicly available due to data privacy protection regulations but are available from the corresponding author on reasonable request.

## Abbreviations

ACP: Advance care planning
EoLC: End-of-Life Care
GP: General practitioner
GP-EoLC-I: General Practice End of Life Care Index
ICD-10: International Statistical Classification of Diseases and Related Health Problems – 10th Revision
IQR: Interquartile range
MRC: Medical research council
OPAL: Optimal care at the end of life
PC: Palliative care
SD: Standard deviation
SPICT™: Supportive and Palliative Care Indicators Tool
SPICT-DE™: Supportive and Palliative Care Indicators Tool, German version
